# Temporal phenotypic responses of early vegetative maize to heat, drought, and combined stresses

**DOI:** 10.1111/tpj.71161

**Published:** 2026-10-10

**Authors:** Anna L. Casto, Katherine M. Murphy, Alejandra L. Quinones, Joshua Sumner, Tess Rogers, Leonardo W. Lima, Malia A. Gehan, Cory Hirsch

**Affiliations:** ^1^ Donald Danforth Plant Science Center 975 N. Warson Rd. St. Louis Missouri 63132 USA; ^2^ Department of Plant Pathology University of Minnesota 1991 Upper Buford Circle St. Paul Minnesota 55108 USA

**Keywords:** maize, *Zea mays*, abiotic stress, high‐throughput phenotyping, image analysis, drought, heat, temperature

## Abstract

Understanding how maize responds to heat, drought, and the combination of stresses is essential for developing resilient cultivars for future climates. We characterized temporal phenotypic responses of 47 genetically diverse *Zea mays* inbred lines grown under control, drought, heat, and combined drought‐heat stresses for 13 days in a high‐throughput phenotyping platform. Plant area was modeled over time using Bayesian hierarchical growth analysis. Treatment explained the largest proportion of phenotypic variance, while genotype × treatment interaction accounted for up to 35% of color variation. Growth and development responses were strongly genotype‐dependent, and combined‐stress responses were more closely related to heat rather than drought responses. Heat primarily reduced height and impacted growth but not development, whereas drought reduced leaf number and greenness and impacted both growth and development, indicating distinct physiological effects. Despite strong genotype effects, subpopulation explained differences only in drought responses. In follow‐up experiments, these early vegetative stresses had a significant impact on days to anthesis and plant area even long after the stresses had stopped. Collectively, these results emphasize that combined‐stress tolerance cannot be inferred from a single‐stress, and that there is potential for improving early environmental stress tolerance that will impact later plant phenotypes.

## Significance Statement

This study integrates temporal image analysis and Bayesian growth modeling to dissect how 47 diverse maize inbred lines respond to drought, heat, and their combination. The results demonstrate genotype and stress‐specific impacts. A follow‐up experiment indicates that early stress impacts days to anthesis and plant area long after the stress has concluded. These findings underscore the need to evaluate maize diversity under multifactorial stress regimes to accurately identify resilient genotypes for environment‐adaptive breeding.

## INTRODUCTION

Maize (*Zea mays* L.) is a staple crop that plays a crucial role in global food security (Erenstein et al., 2022; FAOSTAT, 2024). With a production of 1.2 billion tons in 2023 (FAOSTAT, 2024), maize provides over 20% of caloric intake in emerging economies and serves as a major livestock feed in high‐income countries (Erenstein et al., 2022). Despite century‐long yield gains (Erenstein et al., 2022; Hatfield & Dold, 2018), maize production remains vulnerable to drought, heat, and their combined occurrence (Cohen et al., 2021; Mittler, 2006; Sutanto et al., 2024). In Sinaloa, Mexico, a major maize‐producing region, water limitation lowered yields by 25% on average, heatwaves reduced yields by 10%, and combined drought and heat led to a yield decrease of 44% (Sutanto et al., 2024). Similar yield penalties from drought, heat, and their combination have been reported across other major maize‐producing regions worldwide, indicating that these losses reflect a broad global pattern (Tigchelaar et al., 2018).

Combined drought and high temperatures have increased in frequency, severity, and duration around the globe (Alizadeh et al., 2020; Mazdiyasni & AghaKouchak, 2015; Mukherjee & Mishra, 2021), exacerbating the impact of both of these extreme weather events (Chiang et al., 2018). These patterns are projected to worsen over the 21st century (Tripathy et al., 2023) and affect maize‐producing regions in the United States, Mexico, West Africa, Central Asia, and China (Jägermeyr et al., 2021). Under the most severe scenario, 74% of maize‐producing areas are projected to face negative yield impacts (Jägermeyr et al., 2021). These predictions highlight the need for mitigation efforts to prepare crops to withstand drought, heat, and the combination of stresses. While heat and drought often occur in combination, the presence of irrigation in many countries, as well as lack of water in others, means they also occur as individual stresses. Analyzing phenotypes of diverse germplasm in both individual and combined‐stress treatments will aid in the identification of desirable traits, the varieties that possess them, and molecular mechanisms of stress tolerance to improve a crop's resilience to these environmental stresses (Sato et al., 2024).

Individually, drought and heat impact plant phenotypes through distinct physiological pathways (Cohen et al., 2021). Drought reduces shoot biomass and leaf area (Cohen et al., 2021; Vile et al., 2012), while heat can accelerate development, limit pollen viability, and trigger premature senescence (Cohen et al., 2021; Makore et al., 2021; Vile et al., 2012). Studies on multifactorial stress conditions have found that the co‐occurrence of stresses can have unique responses on plant phenotypes that cannot be attributed to the sum of the independent effects of each individual stress (Sato et al., 2024; Vescio et al., 2020; Zandalinas et al., 2020). Together, these stresses yield non‐additive effects, either synergistic (exceeding the sum of individual stresses) or antagonistic (where one stress partially mitigates the other) (Nadeem et al., 2023; Vescio et al., 2020; Zandalinas & Mittler, 2022). In barley, combined drought and heat stress during the heading stage led to a synergistic reduction in comparison to either individual stress (Mahalingam & Bregitzer, 2019). In maize, heat and drought stress had antagonistic effects on primary root thickness and branching density (Vescio et al., 2020). These differences were attributed to compensatory mechanisms between water deficit and heat stress (Vescio et al., 2020). The intensity of each stress, order of application, and the plant species in question are strong determinants of the type of effect stress combinations will have (Zandalinas & Mittler, 2022). Heat and drought stress have, in particular, been shown as highly detrimental to yield when the stresses occur in the silking, perisilking, and pollen shed stages (Bheemanahalli et al., 2022; Liu, Yu, et al., 2022). While these stages are important, early vegetative stress has the potential to kill the plant outright, or have long‐term effects on plant development, morphology, and potential productivity, and thus early growth stages are the focus of this study. Understanding how the interaction between heat and drought stress manifests across genotypes in vegetative growth stages is critical for breeding programs seeking durable stress tolerance.

Controlled environment chambers enable precise application of abiotic stress and minimize confounding environmental variation (González Guzmán et al., 2022; Langstroff et al., 2022). When coupled with high‐throughput phenotyping technologies (e.g., cameras, controlled watering, RFID tracking), these systems allow for both controlled application of stress treatments and non‐destructive imaging of plant responses (Fahlgren, Feldman, et al., 2015). High‐throughput phenotyping systems have been successfully applied in studies of drought on barley (Chen et al., 2014), setaria (Fahlgren, Feldman, et al., 2015), *Brassica napus* (Ricono et al., 2025), maize (Wu et al., 2021), and combined‐stress treatments on *Brachypodium distachyon* (Ludwig et al., 2023), yet large‐scale, temporally resolved analyses of maize under stress *combinations* remain scarce. Image‐derived traits collected in these systems have been shown to correlate with physiological measurements, such as the strong correlation between plant area and plant biomass (Duenwald et al., 2025; Fahlgren, Feldman, et al., 2015; Fahlgren, Gehan, & Baxter, 2015). However, an important limitation is that these phenotypic measurements are not directly related to physiological mechanisms of plant stress responses, which have been reviewed elsewhere (Tang et al., 2025), but serve as important phenotypic observations and proxies for follow‐up mechanistic studies.

The primary objective of this study is to integrate high‐throughput phenotyping with high temporal resolution for Bayesian growth modeling to quantify the phenotypic responses of diverse maize inbred lines to heat, drought, and combined stress. Here, we leveraged an automated, high‐throughput phenotyping platform to evaluate 47 maize inbred lines, including the NAM founders and diverse WiDiv genotypes, subjected to drought, heat, and combined drought‐heat stress. By integrating daily, image‐based measurements with Bayesian growth modeling, we aimed to quantify how stress treatments alter developmental trajectories, determine genotype‐specific responses, and evaluate whether responses to single stresses can forecast combined‐stress outcomes. We adopted a Bayesian hierarchical growth‐modeling framework, rather than conventional crop growth models such as logistic, Gompertz, or beta‐function models, because it accommodated the non‐sigmoidal growth characteristic of the early vegetative stage, propagated uncertainty through hierarchically structured genotype and treatment effects, and enabled direct probabilistic hypothesis testing of growth‐rate differences between genotypes and treatments. These features are particularly well suited to the daily, image‐based time‐series data generated here (Sumner et al., 2021). Our results reveal distinct and genotype‐specific temporal strategies of maize responses to multifactorial stress, advancing the mechanistic understanding of plant phenotypes under climate‐relevant conditions.

## RESULTS AND DISCUSSION

### Trait variance and selection of traits for temporal analysis

To quantify how maize plants respond to heat, drought, and combined drought and heat stress, we grew 47 diverse maize inbred genotypes under stress conditions for 13 days (days 8 to 20 after planting; Figure 1) in the Bellwether Phenotyping Facility (Fahlgren, Feldman, et al., 2015). We analyzed 25 image‐derived traits across the 47 lines using PlantCV (v3) (Gehan et al., 2017). Variance partitioning using a random‐effects model showed that variance explained by treatment increased as the experiment progressed and was a significant factor in most traits at the end of the experiment (Figure S1b). In addition, genotype and genotype × treatment interactions contributed significantly to several key metrics throughout the experiment (hue circular mean, height, and plant area) (Figure S1a). The interaction term explained up to 35% of variation in hue circular mean, reflecting genotype‐specific color responses to stress at the end of the experiment (Figure S1a). Height and plant area were influenced by both genotype and treatment, indicating that these traits captured physiologically meaningful variation beyond general size effects (Figure S1a).

**Figure 1:**
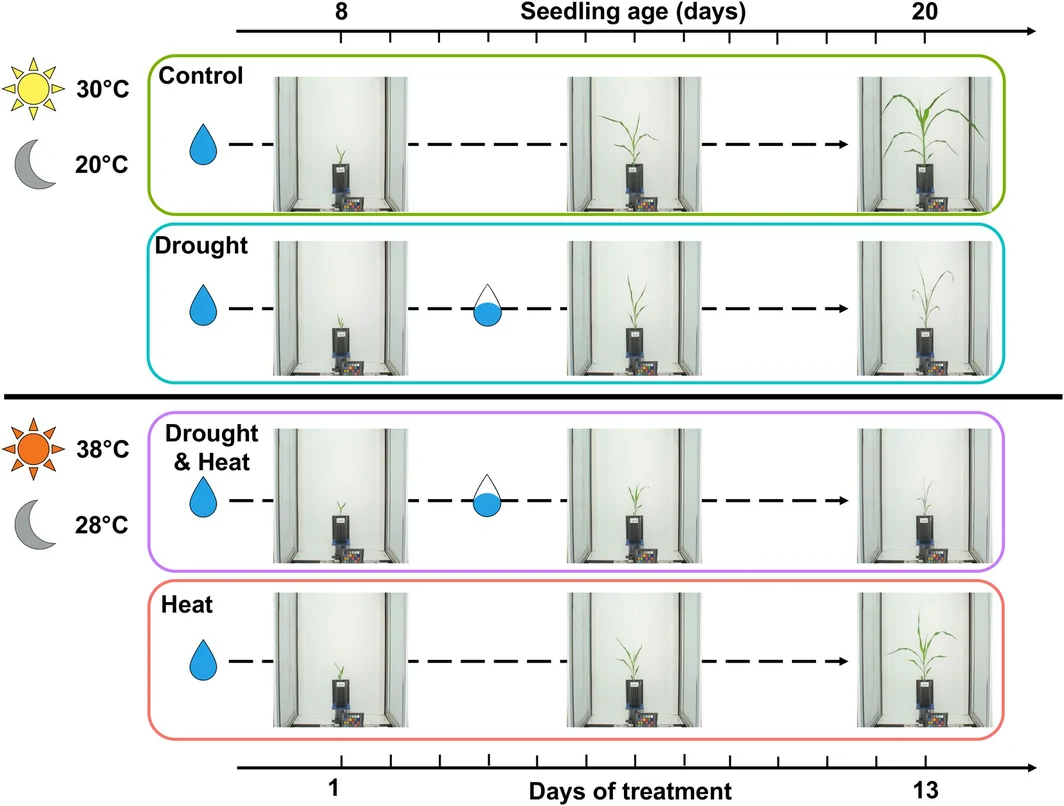
Graphical experimental summary. Plants were grown in a controlled environment set at two different temperatures in two consecutive runs of 13 days each, starting at day 8 after planting. Drought was established as a progressive decrease in water content in both runs. RGB images were collected daily.

Many of the image‐based traits were highly correlated to one another (Figure S2), which is consistent with previous studies (Chen et al., 2014; Duenwald et al., 2025; Wu et al., 2021). Based on this finding, we focused subsequent analysis on plant area, height, and hue circular mean, which jointly represent growth, morphology, and plant color. We also derived pseudo‐water use efficiency (pWUE) as an integrated trait linking morphology with physiology.

### Heat and drought had distinct developmental and growth effects, despite both resulting in smaller plants

In the control treatment, plant size increased exponentially during the experiment, and hue circular mean was stable at ~85°–90° (Figure 2a–c). This hue range falls within the green region of the color wheel, as expected for plants in early vegetative growth stages. In all stress treatments and for all genotypes, plant area and height were lower in stress than in control by the end of the treatment (Figure 2a–c). This result is not unexpected; however, the stress treatments were also not severe enough to cause complete plant death (Murphy et al., 2024; Quiñones et al., 2024). The combined stress had a more severe impact on plant size and color than the individual stresses (Figure 2a–c). This result is consistent with a previous study of both greenhouse and field experiments of maize and several other crop species (Cohen et al., 2021).

**Figure 2:**
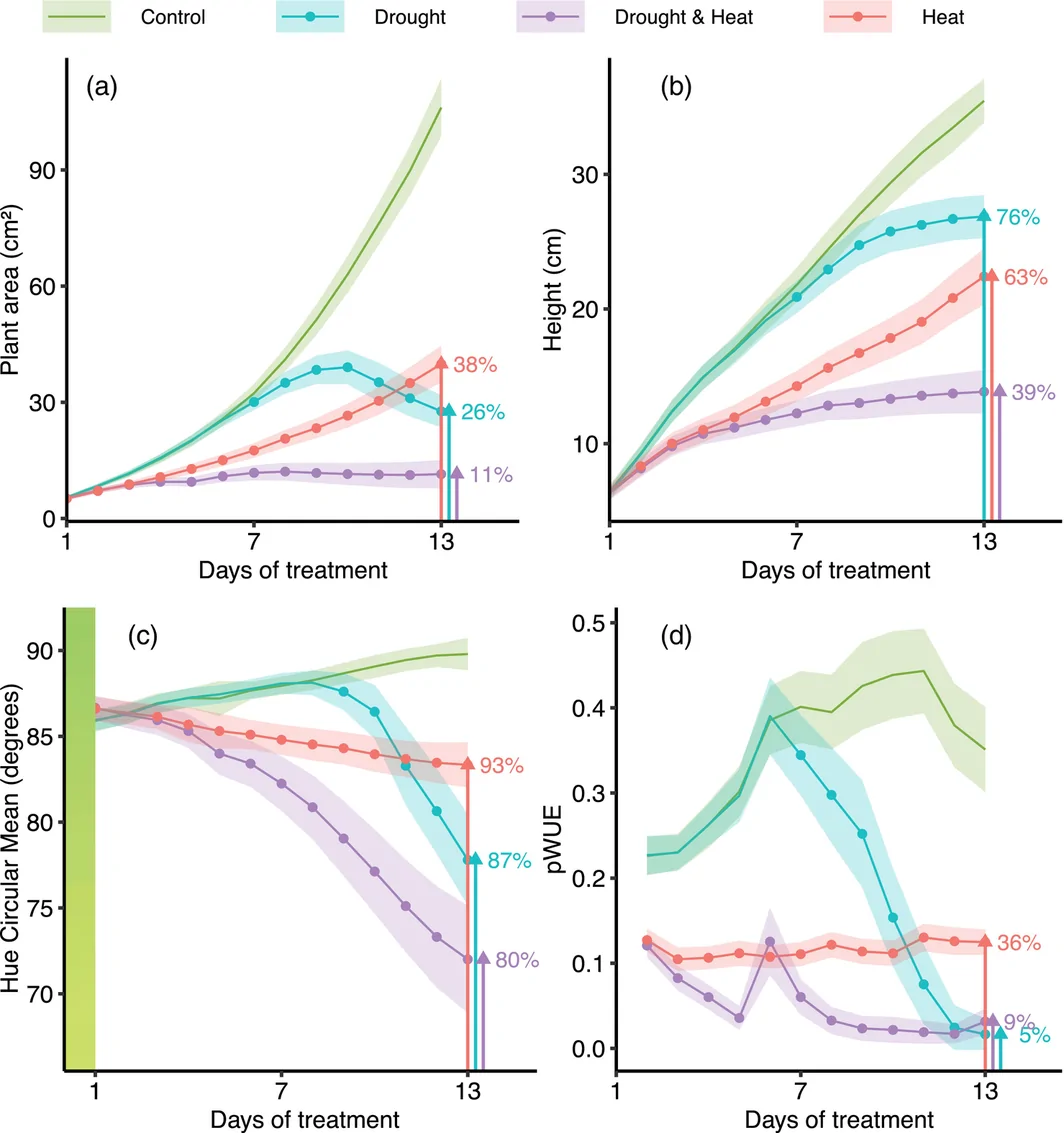
Average trend for each treatment across genotypes. (a) Average area over time. (b) Average plant height over time. (c) Average hue circular mean over time, color bar indicates corresponding plant color. (d) Average pWUE over time. Circle shape points in treatment lines indicate significant Tukey test pairwise comparison to control treatment at each time point (*P* < 0.01); standard error indicated by ribbon. Arrows and percentage values indicate percent similarity to control condition for each treatment at the end of the experiment. Color bar in (c) indicates plant color corresponding to hue degrees.

By the end of the experiment, height was reduced 12.5% (*post hoc* Tukey test, *P* = 2.89 × 10^−13^) more by heat than by drought, while area and hue were 11.5% (*post hoc* Tukey test, *P* = 2.89 × 10^−13^) and 6% (*post hoc* Tukey test, *P* = 2.89 × 10^−13^) more impacted by drought than heat (Figure 2a–c). The plant area of both heat and combined‐stress plants was significantly different from control as early as the end of Day 1, while drought‐stressed plants decreased in plant area only after Day 10 (Figure 2a). This reduction in plant area under drought was not accompanied by loss of leaves; therefore, it is likely caused by leaf rolling, which we observed in the plant images (Figure 1). This has also been reported in previous studies on image‐based phenotyping, where changes in leaf shape reduce surface area and transpiration, and thus decrease plant pixels in the 2D images (Dodig et al., 2021; Shi et al., 2025). It is important to note that this reduction in plant area is not indicative of a change in biomass, and while informative for our purposes of stress response characterization, should be noted as a limitation of plant area as a proxy trait when actual biomass measurements under drought stress are needed.

To understand the effects of stress on plant growth over time, we used Bayesian Hierarchical Modeling in the *pcvr* R package to model plant area (Sumner & Berry, 2025). We fit plant area data to a power law model, which was chosen for its flexibility, given the differences in the growth curves of plants growing under different treatments, particularly under drought stress. Model fit metrics are provided in Table S1. After fitting the data, we used Bayesian hypothesis testing in *pcvr* to test if the growth rate increased or decreased (*B* > 1 or *B* < 1, respectively) for each genotype and treatment. The exponent term (*B*) for plants in control conditions was greater than 1 for all genotypes, indicating an accelerating growth rate over time (Figure 3; Table S2). This is expected because the experiment was done in early plant developmental stages, which is characterized by exponential growth (accelerating growth rate). In contrast, for most genotypes in the combined stress, growth rate slowed over time (*B* < 1, Figure 3). Growth rate accelerated over time (*B* > 1) for 11 of the 47 genotypes in drought stress and 2 genotypes in heat stress (Figure 3; Table S2), but slowed (*B* < 1) for the majority of genotypes under any stress treatment (Figure 3; Table S2). The slowing growth rate in the stress treatments could be attributed to smaller plant area (e.g., smaller leaves) or to delayed development (e.g., fewer leaves).

**Figure 3:**
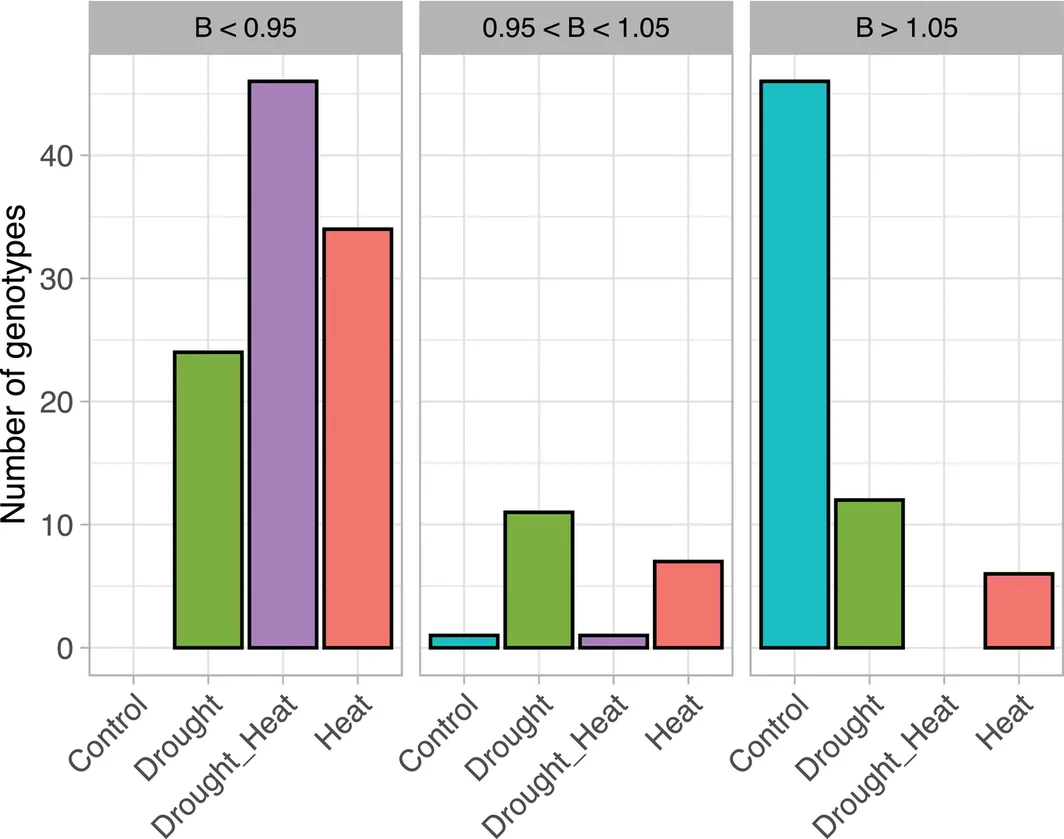
Summary of growth rates across treatments. Counts of genotypes in each treatment with slowing growth (*B* < 1) or accelerating growth (*B* > 1). Plant area over time was fit to a power law model using Bayesian Hierarchical modeling. *B* is the rate term which describes changes in growth rate, where *B* = 1 is approximately linear growth, *B* < 1 indicates slowing growth, and *B* > 1 indicates accelerating growth. Some treatments do not total 47 genotypes because the *B* parameter test for those genotypes was not significant in either category.

To investigate the source of reduced growth rate of the stress‐treated plants, we counted the number of leaves per plant at the endpoint as a proxy for developmental stage, a standard practice for maize (Li et al., 2016). Stress significantly reduced leaf number compared to control for 2 genotypes in heat stress, 28 genotypes in drought stress, and 42 genotypes in the combined stress (Kruskal–Wallis tests with *post hoc* Dunn's test, adjusted *P* < 0.05; Figure S3). This result suggests that while heat stress reduced plant size and growth, it did not delay plant development in most genotypes. On the other hand, reduced size in drought and the combined stress may be partially explained by delayed development for most genotypes (Figure S3). This aligns with prior work showing moderate temperature increases generally accelerate development (Dos Santos et al., 2022; Zhu et al., 2021), whereas drought stress has been shown to delay development (Sah et al., 2020). Of the 28 genotypes that had reduced leaf number in drought stress, only 11 of them had a slowing growth rate (see list of each genotype response in Table S2), demonstrating variation in the timing of growth and development. A plant may have reduced area (smaller leaves) but still develop leaves at the same pace as control, as was the case for most genotypes in heat stress. The combined stress was the most severe and had both reduced growth rates and delayed development (i.e., reduced leaf number).

In summary, these data show that heat and drought have distinct developmental and growth effects, despite both resulting in smaller plants. Heat stress generally reduced growth rate but not developmental stage across genotypes, and drought stress impacted developmental stage for some genotypes but not others. These treatment and genotype‐specific differences in growth rates—accelerating, decelerating, or staying the same as control—may be due to adaptive responses, although additional experiments are necessary to support this hypothesis. Furthermore, identifying these divergent growth dynamics across the diverse germplasm under multifactorial stress conditions advances understanding of stress tolerance beyond single timepoint measurements, and provides a framework for future genetic dissection of stress response mechanisms (Figure 4).

**Figure 4:**
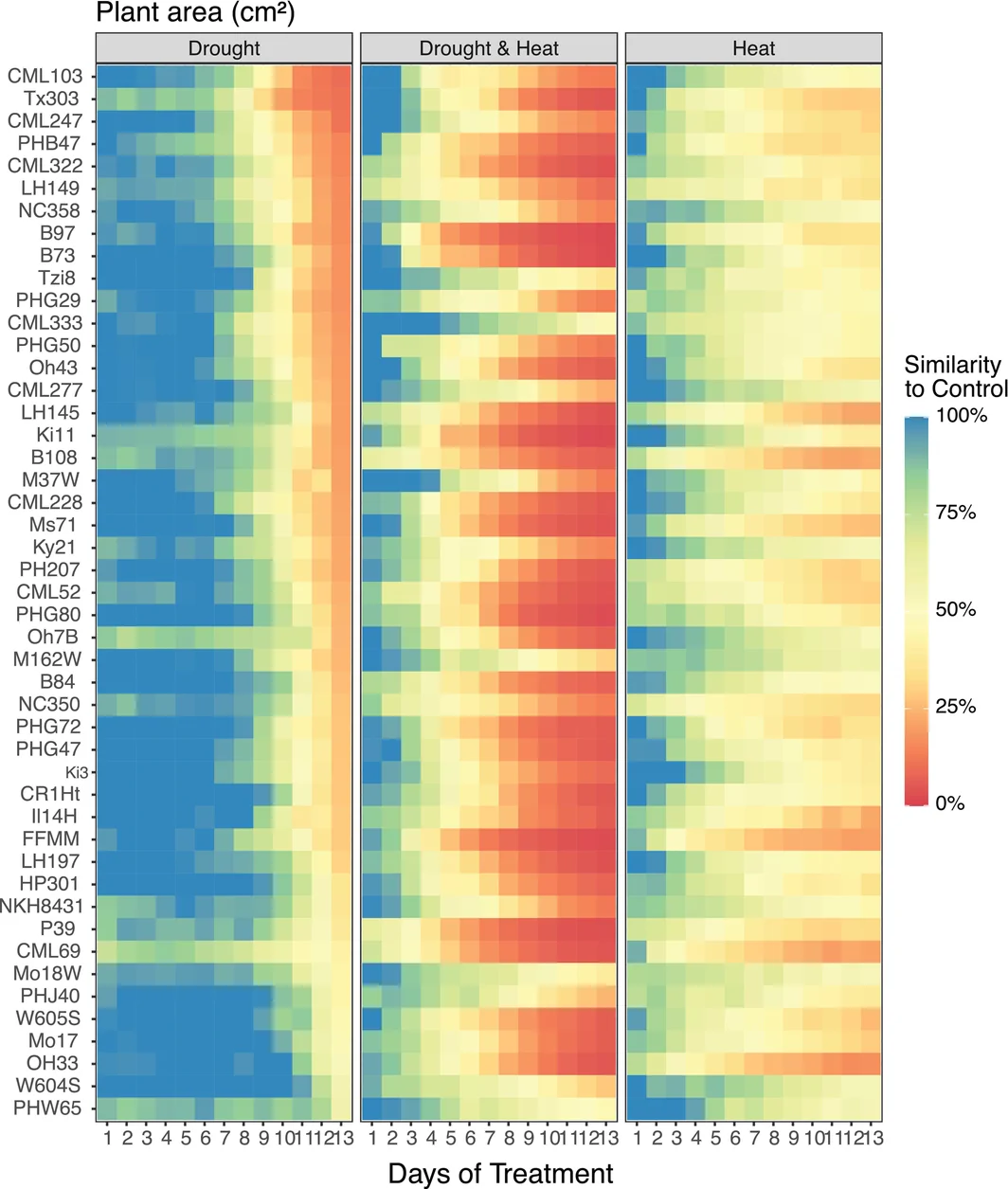
Example of stress response compared to control. Relative change in plant area across time for each treatment for all 47 genotypes.

Bayesian longitudinal modeling revealed new insights into genotype‐specific growth rates in stress responses that would not be detectable from endpoint measurements alone. Using a Bayesian framework enabled fitting non‐linear distributional models allowing for flexible hypothesis testing (Poudel et al., 2024; Sumner et al., 2021).

### Lower pWUE in heat stress was driven by different factors than the combined‐stress treatment

With the distinct relationships between stress treatment, growth, and development, we sought to understand the relationship between plant growth and water use in this experiment. Water used by each plant was calculated daily by weighing the carriage containing the pot, plant, and soil before and after each watering. Pseudo‐water use efficiency (pWUE) was calculated per day using the water used (daily difference between weights on subsequent days) and the image‐extracted plant area as a proxy for biomass, as previously described (Feldman et al., 2018). Therefore, lower pWUE can be driven by a reduction in plant growth (daily change in plant area), an increase in the amount of water used, or both.

In control conditions, total water used and plant area both increased over time, resulting in a stable pWUE from Day 5 to the end of the experiment (0.4–0.45 cm^2^ g^−1^; *P* = 0.065; Figure 2d). We hypothesized that heat and combination‐stressed plants would decrease in pWUE because previous research demonstrated that transpiration increases in heat stress, which leads to evaporative cooling of the leaves, mitigating heat stress (Crawford et al., 2012; Elferjani & Soolanayakanahally, 2018). In support of this hypothesis, plants in heat stress used a similar amount of water to control plants but were much smaller in size, resulting in reduced pWUE (0–0.15 cm^2^ g^−1^) in comparison to control from day one onward (Figure 2d; Figure S4). In the combined stress, reduced water availability and extremely limited growth (i.e., no increase in plant area) resulted in a low pWUE throughout the experiment (0–0.1 cm^2^ g^−1^, Figure 2d; Figure S4). Therefore, though pWUE was similarly low for plants in the heat and combined stresses, the dynamics of water use were different between the two treatments: low pWUE in heat was driven by high water use relative to changes in plant size, but in the combined stress, it was driven by both very slow growth and reduced water use.

In contrast, we hypothesized that drought‐stressed plants would have a higher water use efficiency because plants limit water loss by closing their stomata (Sato et al., 2024). Early in the experiment, drought‐stressed plants had similar pWUE as control plants (*P* = 0.396; Figure 2d). However, pWUE was significantly decreased in comparison to control starting on Day 6 onward (*P* = 9.35e‐05; Figure 2d). This difference in pWUE under drought was driven by a decrease in the daily change in plant area around Day 6–8 onward (*P* = 0.002) while water use also decreased (Figure S4). The decrease in pWUE in drought could indicate that stress response mechanisms, such as closing stomata, resulted in reduced water use and slowed growth or that plants slowed growth by some other mechanism so smaller plants would transpire less, resulting in less water use. It is also possible that leaf curling resulted in reduced projected plant area starting at Day 6, which also reduced the daily change in area. Further experiments are necessary to determine the mechanism behind reduced pWUE in early‐stage drought stress. For example, experiments that closely examine the transcriptome and metabolome under very early drought (Day 5) may help to elucidate these changes in water use dynamics.

### Stress responses in one trait correlated to other trait responses, except for pWUE

We hypothesized that response measured in one trait under a particular stress would correlate to the response measured in other traits in the same stress because of the strong correlation between area, height, and hue (Figure S2). In other words, genotypes most similar to control for plant area in heat would also be most similar to control for height in heat. This is relevant for planning future experiments, because fewer traits could be evaluated if the response of one trait is representative of another trait. The alternative scenario that responses are unique depending on the trait evaluated is also interesting, because it would suggest that plant growth, color, and shape are affected differently in different genotypes. In support of the hypothesis, the area response in each stress had a strong positive correlation to the height response for the same stress (Table S3, Spearman's Correlation = 0.63–0.75, *P* < 0.001). This suggests that either trait is suitable for measuring plant stress responses in future experiments in this species and phenotyping system. We expected a similar relationship between plant area and hue responses due to the correlation between the traits, and because chlorosis (yellowing) is an indicator of poor plant health and reduced photosynthesis. Although the relationship was weaker, the area response was correlated to the hue response within each stress treatment (Table S3, Spearman's Correlation = 0.36–0.72, *P* < 0.001).

Given that pWUE is calculated using changes in plant area, we also expected the pWUE response to correlate with the plant area response. Consistent with this hypothesis was a significant correlation between area response, height response, and the pWUE response to the combined stress (Table S3, Spearman's Correlation = 0.72 and 0.51, respectively, *P* < 0.001).

However, plant area response was not significantly correlated to pWUE response for drought (*P* = 0.82) or heat (*P* = 0.27) treated plants. In other words, plants that maintained size compared to control did not necessarily have higher or lower pWUE in the stress condition. Under drought, this decoupling has been seen elsewhere, and others have considered that size reduction can itself represent a water‐conservation strategy rather than a consequence of low efficiency, and improvements in WUE under drought conditions are often achieved at the cost of reduced biomass accumulation, and thus result in lower biomass and yield (Blum, 2009; Claeys & Inzé, 2013). Under heat stress, a different physiological mechanism may contribute to the lack of WUE–area correlation: higher temperatures progressively decouple stomatal conductance, transpiration, and photosynthesis, as stomata open to facilitate transpirational leaf cooling, reducing WUE independent of growth responses (Diao et al., 2024). Overall, these results suggest that pWUE might not correlate with larger plant size under drought or heat, and conflicts with reports that increasing pWUE in drought positively impacts plant biomass (Zhao et al., 2018). However, the relationship between WUE and growth is highly context‐dependent, as drought response is largely determined by growth stage, drought severity, and the duration and pattern of water deficit (Claeys & Inzé, 2013; Tardieu, 2012).

### Plant traits in control were not predictive of stress responses

Since there were correlations between plant responses within a stress (with the exception of pWUE), we were interested to see if phenotypes in control conditions could predict stress responses. Strong correlations between control phenotypes and phenotypes under one or more stresses would allow researchers to predict a plant's response to abiotic stress using only control phenotypes, reducing the number of conditions a researcher would have to test.

Plant area in control conditions was weakly, negatively correlated to the area response in drought (Table S4, ⍴ = −0.42 *P* = 0.006), suggesting that plants that were larger in control conditions had a greater size reduction in drought stress. Control hue was negatively correlated with hue in heat stress (Table S4, ⍴ = −0.80 *P* < 0.001), suggesting that greener control plants had a greater change in color in heat stress. While a body of work has found that heat stress reduces chlorophyll content (Djalovic et al., 2024), the finding that genotypes that are greener under control conditions (in other words, baseline greenness) show a greater change in hue is less well explored in the literature.

We found no other significant correlation between plant area, height, or hue under control conditions and the change in the trait due to the three stress conditions (Table S4). This suggests that, beyond the limited case of plant area in drought and control hue in heat stress, plant size and color in control conditions do not generally predict stress responses. Other researchers found that the phenotypes they found that were correlated to yield were different in well‐watered and drought stress conditions (Cairns et al., 2012). Like our results, this suggests that plant phenotypes should be measured in the environments for which they are intended to grow for successful breeding; this is also supported by the large body of literature on genotype × environment (G × E) interactions where different genotypes perform differently in different environments (Messmer et al., 2009).

### Subpopulation was a significant factor in drought but not heat responses

While traits under control conditions were not generally predictive of stress responses, we were interested to test if maize subpopulations responded to stress similarly. Subpopulations are groups of genotypes with shared breeding history, genetic similarity, and/or geographic origin (Li et al., 2022). Within the subpopulations used in this study, temperate subpopulations, like stiff‐stalk and non‐stiff‐stalk, have been historically selected for tolerance to common abiotic stresses such as low soil moisture, low nitrogen, and cold (Revilla et al., 2016; Tollenaar & Wu, 1999), as compared to tropical lines (Sun et al., 2023). Assuming that this historical selection for abiotic stress tolerance means that temperate lines are less impacted by drought stress, we hypothesized that stiff‐stalk and non‐stiff‐stalk subpopulations would also be less responsive (more similar to control) to the treatments than tropical lines in our data. To test this, stress responses of tropical, stiff‐stalk, and non‐stiff‐stalk genotypes (13, 9, and 12 genotypes, respectively) were compared for each treatment and trait using beta regressions (see Materials and Methods). Likelihood ratio tests were used to compare regressions with and without subpopulation as a factor to determine its significance. Subpopulation was a significant factor for plant area and height under drought stress (*X* ^2^ = 6.8, *P* = 0.03; *X* ^2^ = 6.24, *P* = 0.04; Figure S8), but not heat or the combined stress. To understand these changes we compared average responses to stress in each subpopulation. Temperate subpopulations were more similar to control than tropical subpopulations for plant area (non‐stiff‐stalk different from tropical, *P* = 0.02) and height (stiff‐stalk different from tropical, *P* = 0.04), consistent with their breeding history in low‐rainfall environments (Tollenaar & Wu, 1999). The two temperate groups (stiff‐stalk and non‐stiff‐stalk) were not different from each other for these traits (*P* > 0.05).

Explaining the absence of a subpopulation effect in heat stress responses is more complex: prior studies have shown a trade‐off in moderate and severe heat stress tolerance in historic breeding populations (Kusmec et al., 2023), suggesting shared breeding history does not necessarily lead to similar temperature stress responses. The different influences of subpopulation on drought and heat response are reflected both in the literature and in our own data. Phenotypes under drought or heat as individual stresses were not correlated in this experiment or in others (Malenica et al., 2021; Nelimor et al., 2019), suggesting unique response mechanisms. These findings suggest that, for the population and growth stage tested here, variation in drought stress response could be due to a shared breeding history targeted for drought tolerance for temperate subpopulations (Tollenaar & Wu, 1999). However, we do not see the same relationship between subpopulation and heat stress, which have not had the same breeding targets for extreme heat stress (Kusmec et al., 2023), but testing more germplasm may provide additional insight. Additional genotypes and larger subpopulation groups would need to be evaluated to appropriately attribute genetic determinants of the phenotypes measured here and compare additional subpopulations.

### Timing of stress onset was related to the magnitude of response in plant height but no other traits

Subpopulation was found to be important for drought stress but no other stresses, and plant phenotypes under the control conditions generally did not predict stress response. However, neither of these questions take into account the temporal dynamics of stress response. We hypothesized that genotypes with earlier detectable stress phenotypes would also have a greater difference from control at the end of the experiment. To test this, Bayesian hypothesis testing and growth models were used to determine the earliest day the stress effect could be detected (Figure 5; Table S5). For both heat and the combined‐stress treatments, most genotypes had a significant difference from control on Day 5 (Figure 5). The two genotypes that had later stress onset in heat and the combined stress were not the same genotype, but were both tropical lines (CML333 and CML228).

**Figure 5:**
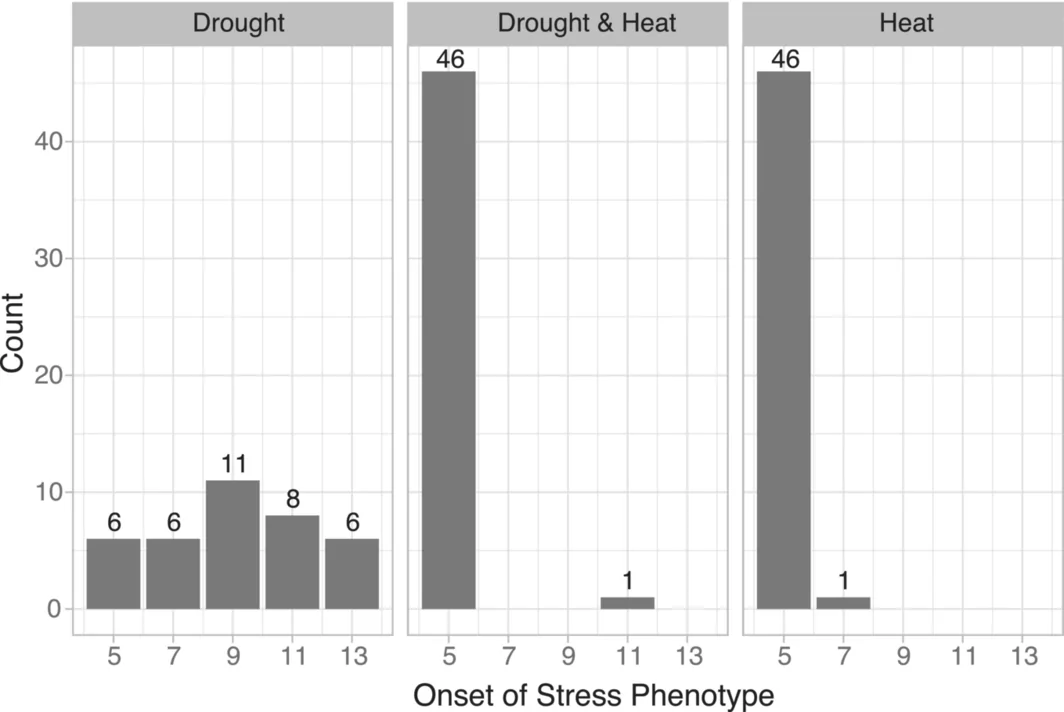
Day of stress onset. Counts of genotypes on the first day that plant area is significantly different from control in each treatment.

Genotypes in the drought treatment showed greater variation in the day of stress onset than heat or combined stresses (Figure 5). Therefore, we focused our hypothesis test on the drought treatment to determine if earlier detectable phenotypes would also have a greater difference from control at the end of the experiment. We correlated the day of stress phenotype detection to the endpoint stress response for each trait (Table S6). Height response at the end of the experiment was weakly, positively correlated with the day of stress phenotype onset (Spearman's Correlation = 0.33, *P* = 0.047, Table S6). This supports our hypothesis that early detection of a stress phenotype results in a greater response. However, no other phenotype responses (area or hue) showed significant correlations with stress phenotype onset day. These data indicate that there are genotype‐dependent differences in early detection of drought, but that those differences are only predictive for the height response. Designing an experiment, therefore, to simply find the first detectable change in a trait is not informative for finding the genotype that shows the greatest response after the end of a stress period. Furthermore, while single timepoint measurements are common due to restrictions of labor and cost, temporal modeling provides significant additional insight into plant stress responses and holds potential predictive insights, depending on the trait.

### Combined‐stress responses were most similar to heat stress responses

Drought and heat stress often occur together, so we hypothesized that genotypes that were more responsive to drought would also be more responsive to heat. However, the response of traits in drought stress was not correlated to response in heat stress (Table S3), suggesting some genotypes will perform better in one stress condition than the other. Despite their typical co‐occurrence in nature, response to drought was not predictive of response to heat, and vice versa, within the conditions tested here (Figure 4; Figures S5, S7). This is particularly relevant when determining which genotypes to plant in growing areas that may have abundant irrigation but higher temperatures, or the opposite scenario (Cairns et al., 2013).

We also hypothesized that individual responses would be correlated to the combined‐stress response. In support of this hypothesis, the response of plant area and height in the combined stress was strongly correlated to plant area and height responses in heat stress when comparing genotype responses (Spearman's Correlation = 0.68, *P* < 0.001 for area, Spearman's Correlation = 0.56, *P* < 0.001, respectively). However, there was no relationship between drought stress responses and combined‐stress responses for any phenotypes (Table S3). Thus heat, but not drought, may be predictive of plant responses to the combined stress. This is consistent with previous literature in maize. For example, Nelimor et al. (2019) concluded that heat‐tolerant genotypes were tolerant to the combined stress because grain yields under heat and combined stress were correlated. Nelimor et al. (2019) also found that genotypes tolerant to the drought stress were not correlated to ones tolerant to heat or the combined stress. In addition, the transcriptomic and phenotypic responses of maize and other species are unique in the combined stress and are not simply a blend or sum of the individual stresses, suggesting that tolerance to one stress would not necessarily result in tolerance to their combination (Rizhsky et al., 2002; Sato et al., 2024; Vescio et al., 2020; Zandalinas et al., 2020).

Given that heat stress began immediately upon the start of treatment, but drought stress requires a progressive reduction in soil moisture over time, it is perhaps not surprising that heat, rather than drought, was correlated to the combined‐stress phenotypes. We therefore interpret the closer resemblance of the combined‐stress response to heat with caution: because heat stress was imposed immediately at 38°/28°C whereas drought developed progressively through declining soil water availability, this relationship may partly reflect the more rapid application and different temporal dynamics of heat stress rather than an inherently stronger contribution of heat itself.

### Combined stresses generally had a synergistic effect

Combinations of two or more stresses can produce a unique effect from each stress alone depending on the interactions between them (Sato et al., 2024). Interactions between co‐occurring stresses have been described as equal to (additive), greater than (synergistic), or less than (antagonistic) the sum of each individual stress (Zandalinas & Mittler, 2022). The importance of interaction classification lies in its potential for pointing at the specific molecular pathways underlying the observed phenotypes. A classic example of this is the antagonistic effect of combined drought and heat on stomatal opening, which was found to be caused by the prioritization of drought‐responsive molecular pathways despite the presence of heat, which resulted in the elevation of leaf temperature to harmful temperatures (Zandalinas & Mittler, 2022). Similarly, we propose that the classification of the effect of combined stress in the response traits could point toward genotypes with interesting genetics that could be useful in future studies looking into the molecular basis of combined‐stress tolerance.

To study the interaction between drought and heat in our phenotypic responses, we used a multiplicative risk model to calculate the predicted additive effect of the combined stress (Darling et al., 2010; Vescio et al., 2020; see Materials and Methods) and compare it to the observed combined‐stress effect on the final day of treatment (Figure 6a). The combined stress generally had a synergistic effect in most genotypes for plant area and height, an antagonistic effect for hue, and an additive effect for pWUE (Figure 6b). In other words, for maize at an early stage of development, heat and drought stress result in an equal or more severe effect on pWUE and plant size than either of the individual stresses. The antagonistic effect of the combined stress on hue may be due to the complexity of changes in plant color during stress. For example, under drought stress conditions specific genotypes of *B. napus* become a darker shade of green (higher hue value) or turn yellow to brown if leaves senesce (Ricono et al., 2025).

**Figure 6:**
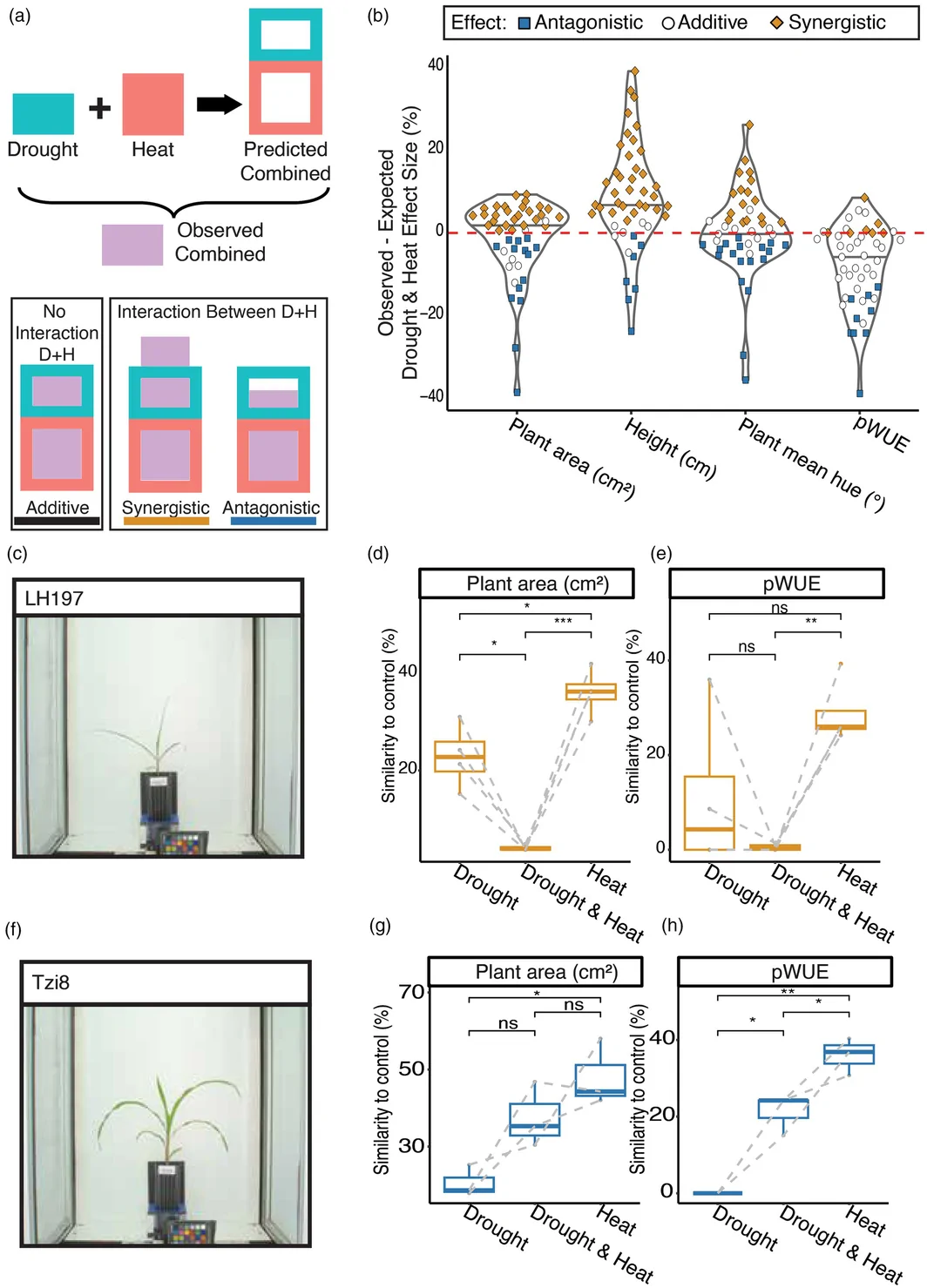
Effect of combined stress across traits. (a) Visual summary of effect classifications. (b) Combined‐stress effect category in each trait across all genotypes, as determined by multiplicative risk model. (c) LH197 plant under combined stress. (d) Plant area and (e) pWUE for the four genotypes with synergistic effects across all traits. (f) Tzi8 plant under combined stress. (g) Plant area and (h) pWUE for the three genotypes with antagonistic effects across all traits.

Despite these general trends, several genotypes differed in their responses to combined stress. In four genotypes (CML228, CML322, CML52, and LH197), the combined stress had a synergistic effect on all phenotypes, suggesting the combined stress had an amplified effect on them (Figure 6c–e). Based on this, we would predict that these genotypes would grow more poorly in environments with both stresses present. On the other hand, in three genotypes, CML333, M162W, and Tzi8, the combined stress had antagonistic effects across all traits. In these genotypes, drought stress alone had a greater impact on plant area, hue, and pWUE than the combined stresses (Figure 6f–h; Figure S6). For these genotypes, we hypothesize that heat stress induces stress response pathways that prime plants for drought stress, which generally had a later onset as an individual stress (Figure 5). Genotypes that differ from the general trends in which the effect of combined stress was antagonistic would be worth examining more closely in future experiments.

### Early vegetative stress had long‐term effects on plant size and flowering time

In this experiment, early stress exposure had significant effects on plant size and color, but it was unclear what the long‐term effects of this stress would be once plants were returned to control growth conditions. We hypothesized that the 2‐week long early vegetative stresses would affect later vegetative and reproductive phenotypes, even when plants are returned to control conditions. To investigate these effects, we repeated the experiment with B73 and Mo17 in the same 2‐week stress conditions, followed by control conditions until anthesis. Both genotypes had a significantly greater number of days to anthesis after drought treatment (*P* < 0.001) and reduced days to anthesis after heat stress (*P* < 0.05, Figure 7a). The combined stress increased the number of days to anthesis in B73 (*P* < 0.001), but did not affect anthesis in Mo17 (Figure 7a). These results demonstrate that stress exposure early in development significantly impacts the reproductive stage in maize. This is consistent with previous research, where higher temperatures have been found to accelerate anthesis (Lizaso et al., 2018), and drought found to delay it (Sah et al., 2020). The persistence of the effect from early to late development, including priming effects for later stress events, has also been explored elsewhere as somatic (also called intragenerational) stress memory (Liu, Quan, & Bartels, 2022). These results, and the prior literature, suggest that there is also significant value in future field experiments that further test the impact of early stress on yield.

**Figure 7:**
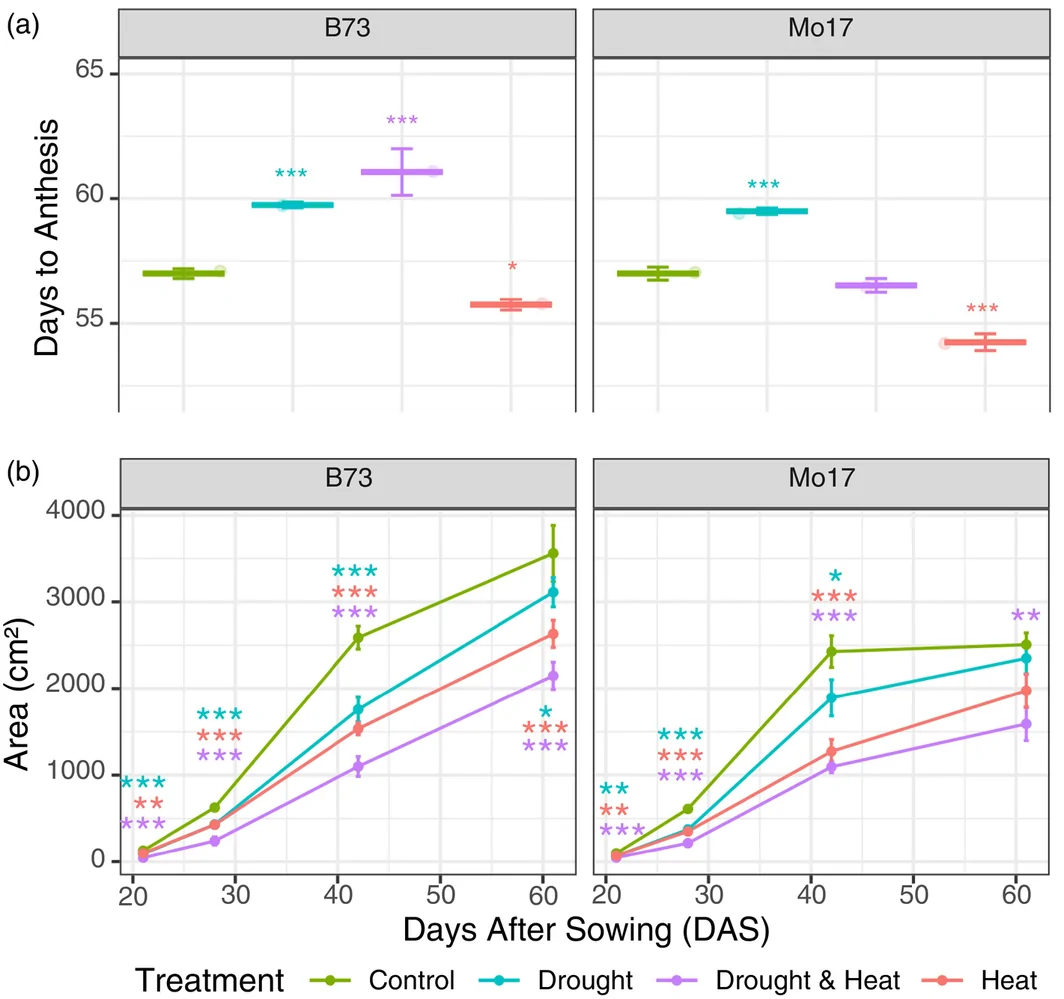
Early‐stage stress impacts later stage phenotypes. (a) Days to anthesis was affected by early stress treatment compared to control conditions. (b) Plant area is reduced by stress treatment when returned to control conditions. *P < 0.05, **P < 0.01, ***P < 0.001.

Next, we evaluated the long‐term effects of stress on plant size by measuring plant area at the end of the 2‐week stress treatment and at three subsequent timepoints after the plants had been returned to control conditions. All three stress treatments significantly reduced plant area at 28 and 42 days after planting (Figure 7b). By 61 days after planting, plants that had been treated with the combined stress still had significantly reduced plant area in both genotypes (*P* < 0.01), and drought and heat‐treated B73 plants were still significantly smaller (*P* < 0.05, Figure 7b). However, drought and heat‐treated Mo17 plants recovered to control plant area by Day 61 (Figure 7b). These results suggest that water availability and temperature are potentially opposing cues for developmental speed, and explain why the combined stress had no change in anthesis compared to control for Mo17. These results further suggest that the effects of early abiotic stress persist through later growth stages, and do so in a genotype‐dependent manner.

## CONCLUSIONS

Heat, drought, and their combined stresses have severe impacts on plant growth, health, and reproduction at many life stages, reducing yields and thus threatening global food security. Here, we show that early individual and combined stresses reduce plant size, greenness, growth rates, and water use efficiency, but the effects of the individual and combined stresses are unique. Despite their co‐occurrence in nature, drought and heat responses did not correlate. Further, while both stresses impacted growth rates in some genotypes, only drought delayed plant development.

Phenotypes of control plants were not predictive of stress responses, suggesting different stress conditions must be tested to understand these responses and develop climate resilient varieties. Additionally, earlier onset of a stress did not influence the endpoint severity of the stress response. Therefore, researchers should be cautious not to discard the genotypes with early drought phenotype onset because they are not necessarily the most different from control by the end of the stress period. These results demonstrate the importance of temporal phenotyping made possible through non‐destructive, image‐based measurements. Furthermore, while a significant body of work has shown that drought and heat stress have significant impacts when applied at the reproductive stage (Bheemanahalli et al., 2022; Liu, Yu, et al., 2022), here we have also shown that even early‐stage, non‐lethal stress also has significant impacts on later growth and reproduction. Together, these results demonstrate the need and value of understanding stress responses that occur early in development to improve plant resilience and productivity.

## MATERIALS AND METHODS

### Germplasm selection

A total of 47 inbred maize genotypes were selected for this study, including the 25 Nested Association Mapping (NAM) inbred parents and Mo17 (Chen et al., 2023; Fattel et al., 2024; Gage et al., 2020; Hufford et al., 2021; McMullen et al., 2009), and 20 lines unique to the Wisconsin Diversity (WiDiv) panel (Hansey et al., 2011). Fast‐flowering mini maize (FFMM) was also selected for its utility in plant science research, but it should be noted that it has a shortened lifespan compared to the other varieties (McCaw et al., 2016). The selection of maize lines aimed to maximize genetic and phenotypic diversity and thus included lines from diverse countries of origin, maturity groups, and subpopulations (Table S7). Subpopulations represented in these germplasm included iodent, non‐stiff‐stalk, popcorn, stiff‐stalk, sweet corn, temperate/tropical mix, tropical, specialized (FFMM), and unknown (B108; as previously labeled by Renk et al., 2021). Additional information on these varieties, including previously collected phenotyping information from other growth stages, is available in (Hansey et al., 2011).

### Plant growth and stress treatments

A total of 24 seeds per line were individually planted ~1 cm deep in 98‐cell plug trays containing Berger BM2 potting soil, which contains no starter charge of nutrients. Pots were watered with reverse osmosis water (all soil materials from Hummert International). Plants were grown in a controlled environment growth chamber (Conviron, BDW80) at 30°C day and 20°C night temperatures, 60% relative humidity, 16 hours daylength, and 400 μmol m^−2^ sec^−1^ light intensity using LED light source ~0.75 m from the tray. Five days after planting, seedlings of uniform size by genotype were transplanted to 950 ml pots (MT38 Stuewe and Sons tree pots). Six replicate seedlings were selected for each line and treatment, except B73, which had nine replicates, totaling 18 seedlings of B73 and 12 seedlings of all other genotypes. Transplanted plants were returned to the same environmental conditions as during germination for another 2 days. Transplant pots contained a 1:1 mixture by volume of Berger BM2 potting soil and Turface MVP pre‐mixed with 3 lbs/cubic yard of Osmocote Classic 14‐14‐14 NPK slow release fertilizer. Each pot was labeled with a custom barcode for the line, treatment, and replicate number.

Seven days after planting, blue mesh screens (Duck Select Grip EasyLiner, cut to pot size) were added to the top of each pot to reduce soil evaporation and improve image segmentation. The pots were then loaded into Bellwether Phenotyping Facility at the Donald Danforth Plant Science Center on individual carriers containing RFID chips. The barcodes were associated with the RFID chips on each carrier to encode the imaging, rotation, and watering schedule, as previously described (Fahlgren, Feldman, et al., 2015). Plants were watered automatically based on pre‐set weights, and plants were weighed before and after watering each night. Genotypes, treatments, and replicates were randomized by loading one replicate of treatment for one genotype onto the system at a time, ensuring a distribution of genotypes, replicates, and treatments throughout the growth house. Following watering, the position of each plant was rotated 15 carriages ahead (half a lane) in order to reduce edge effects in the growth chamber, which further randomized the order and location of the carriages. Plants were imaged in the order of their carriage location daily, ensuring that treatments, genotypes, and replicates were spread across the imaging window.

The controlled environment growth chamber of the Bellwether Phenotyping Facility can only be set to a single day/night temperature schedule, so our experiment was carried out as two runs. For the first run, the growth chamber in the Bellwether Phenotyping Facility maintained the control growth conditions (30°C day and 20°C night), and the external room containing the imaging loop that the plants enter once per day was set to a constant 29°C. For the second run, the growth chamber was set to heat stress conditions of 38°C day and 28°C night. The external room was set to a constant 30°C.

Drought stress was applied to plants in both runs (drought‐only and the combined drought and heat treatments) by gravimetric measurements. Plants were weighed individually each night and watered with reverse osmosis (RO) water to a target weight, as previously described (Fahlgren, Feldman, et al., 2015). In order to determine target weights prior to the experimental runs, four pots containing the same soil mixture were weighed dry and fully saturated with water, and the field capacity was calculated as:

$$\text{Field capacity}\;\left(\%\right)=\frac{\text{Water content}\;\left(\mathrm{ml}\right)}{\text{Saturated water content}\;\left(\mathrm{ml}\right)}\times 100$$

The target weight for each day was calculated as the total weight of the carrier, saucer, dry pot, and water weight necessary to reach the target field capacity. This calculation does not account for the growth of the plant, so the target field capacity was increased by 31 g for control and heat‐stressed plants in the last three nights on the platform, which preliminary work has shown to be the average weight of control plants at 21 days after planting. The target field capacity used for well‐watered (control and heat) plants was 80%. Target field capacity for the drought treatment was reduced stepwise from 80% on Days 1 and 2 to 70% on Days 3 and 4 and 60% on Days 5 and 6, after which soil moisture was allowed to progressively decline from 69% on Day 7 to 44% by Day 14 (Day 7: 69%, Day 8: 65%, Day 9: 60%, Day 10: 56%, Day 11: 52%, Day 12: 49%, Day 13: 46%, Day 14: 44%). Actual soil moisture before and after watering is provided in the analysis scripts on GitHub. This difference in the timing and intensity of drought relative to the immediate imposition of heat is considered when interpreting the combined‐stress response (see Results and Discussion).

The experiment was repeated a second time with B73 and Mo17 to determine the effects of early stress on long‐term plant growth. To do so, plants were grown and stressed in identical conditions and growth chambers described above. On the last day of stress (20 days after planting, DAP), plants were imaged in the Bellwether Phenotyping Facility, then transplanted to 2 gallon (7.57 L) pots with BM7‐35% soil, and top‐dressed with 0.66 tsp (~3.3 ml) of Osmocote+ 15‐9‐12 (NPK) and 0.66 tsp (~3.3 ml) Sprint 330. Plants were grown in a greenhouse with 16 hour day length, 28°C day/22°C night, minimum 40% relative humidity, and supplemental LED lights when the sunlight at the plant level was below 600 W m^−2^ during daytime hours. Plants were watered daily, using Jack's 15‐5‐15 Ca‐Mg diluted to 200 ppm nitrogen on weekdays and industrial water on weekends. Plants were imaged using a Nikon D600 camera as previously described at 28, 42, and 61 DAP (Duenwald et al., 2025). Most control plants had begun anthesis by 61 DAP, so imaging was concluded on this day.

### Imaging and image analysis

Plants were automatically imaged daily using a ⅔ progressive scan CCD camera (Basler AG, Ahrensburg, Germany) with a resolution of 2454 × 2056 pixels for RGB (red‐green‐blue) images. Plants were rotated to capture two angles (0° and 90°) and imaged from the side view. Raw images and associated metadata are available in the public repository Bioimage Archive (Hartley et al., 2021; Sarkans et al., 2018) and linked at https://github.com/danforthcenter/maize‐abiotic‐stress‐paper‐2026.

Images were analyzed using the python package PlantCV v3 (Gehan et al., 2017). All scripts and extracted data are available on GitHub (https://github.com/danforthcenter/maize‐abiotic‐stress‐paper‐2026). Images were color corrected with a log transformation in order to provide a linear color scale from black to white, followed by an affine color correction using a standard color card (Calbrite ColorChecker Passport). Naive Bayes segmentation was used to generate binary masks from each image. The Naive Bayes model was trained on RGB values from 7047 pixels from a sample of 20 images. Masks were cleaned up using the *fill* function. A region of interest was used to isolate only plant objects in the images. A horizontal line was set as a marker for the plant‐pot boundary. The *analyze_size*, *analyze_bound_horizontal*, and *analyze_color* functions from PlantCV were used to extract trait data from each image. For area, width, and height measurements, the maximum value between the two image angles was used for downstream analyses. This ensured that we captured the widest face of the maize plants when measuring each trait. For height, the trait *height_above_reference* was used to ensure height was measured from the horizontal line marking the base of the plant. Hue circular mean measurements from the two angles on each day were averaged for each replicate. Image analysis scripts are available on GitHub (https://github.com/danforthcenter/maize‐abiotic‐stress‐paper‐2026).

### Statistical analysis

All data curation and statistical analyses were performed in R (R Core Team, 2021).

Two filters of 3 standard deviations using all values recorded throughout the experiment were calculated for the plant area and circular mean hue traits. Based on these, a minimum threshold of 300 pixels and maximum of 200 000 pixels was applied to plant area, and hue circular mean values below 25° (red) were removed. Data points considered outliers by either filter were removed from the whole dataset on that day.

To evaluate the significance of subpopulation as a factor influencing response to stress, beta regression models with and without subpopulation as a factor were run and compared through a likelihood ratio test. This was done to account for the beta distribution of the ‘response’ factor: bound between 0 and 100%. All code is available on GitHub (https://github.com/danforthcenter/maize‐abiotic‐stress‐paper‐2026).

### Calculation of pseudo‐water use efficiency (pWUE)

Watering data and image‐derived plant area were used to calculate pseudo‐water use efficiency (pWUE) with the R package *pcvr* (Sumner & Berry, 2025). pWUE, as defined here, uses plant area as a proxy for biomass and is an estimate of vegetative growth efficiency and does not reflect final yield‐based water use efficiency. pWUE was calculated using the following equation:

$$\text{pWUE}=\frac{\text{Δplant area}\;\mathrm{per}\;\mathrm{day}}{\text{Water used}\;\mathrm{per}\;\mathrm{day}}$$

where Δplant area is the difference in projected plant area from 1 day to the next and water used is the difference in pot weight after watering on 1 day and before watering the next day.

### Growth modeling

Plant growth was modeled for each accession and treatment group using Bayesian Hierarchical modeling in *pcvr*. Our data variables were plant area (cm^2^) and time, which was expressed as experimental day. Plant area was fit to a power law model, which is described by the following formula, where *A* is the scaling factor, *B* is the rate term, and *x* is the experimental day.

$$\text{Plant area}\;\left({\mathrm{cm}}^{2}\right)\sim T\left(\mathrm{mu},\text{sigma},\mathrm{nu}\right)$$

$$\mathrm{mu}\sim A*x^{B}$$

$$\text{sigma}\sim a/\left(1+e^{\left(\left(b-x\right)/c\right)}\right)$$

nu~Genotype

A~Genotype

B,a,b,c~Genotype+Treatment

Priors:

$$A=\text{LogNormal}\left(\log\left(3\right),0.25\right)$$

$$B=\text{Normal}\left(1,0.25\right)$$

$$a=\text{LogNormal}\left(\log\left(3\right),0.25\right)$$

$$b,c=\text{LogNormal}\left(\log\left(5\right),0.25\right)$$

$$\mathrm{nu}=\text{Normal}\left(2.7,0.8\right)$$

This was a distributional model using the Student T distribution and thus includes priors on the growth model parameters, parameters controlling variance, and the degrees of freedom. Priors were mostly lognormal distributions since the area phenotype is confined to be positive, with Normal distributions used for degrees of freedom and the rate term. The rate term was constrained to be positive by truncating the prior at 0 and was centered on 1 to assume linear growth while allowing for increasing or decreasing rates of change as informed by the data. The logistic formula for sigma shown above was used in early stress detection models to make fitting simpler with less total data but in the complete models used for the main conclusions a spline was used to model variance instead, additionally the prior on B was LogNormal(log(2.5), 0.25) for the complete models and was also changed to account for having less data in early timepoint models. The model assumed monotonically increasing growth. All priors and further details and the code to implement our models are on GitHub: https://github.com/danforthcenter/maize‐abiotic‐stress‐paper‐2026. Additional models were considered and compared with WAIC (Widely Applicable Information Criterion) and LOOIC (Leave‐One‐Out Information Criterion) (see GitHub), further identifying the power law as the appropriate model choice. Prior predictive checks were conducted using the plotPrior function from the pcvr R package (Sumner & Berry, 2025) (Figure S9). These models were fit in R using the brms (brms version 2.20.4, cmdstanr version 2.34.0) and pcvr packages (version 1.2.1.0) to call Stan version 2.32.2. Models were fit with 4 chains and 2000 post‐warmup draws, early stress detection models were fit using data up to Day 4 then again adding 2 days of data at a time until using data up to Day 12. Bulk and tail effective sample sizes based on MCMC range from 700 to 2200 with an average neff ratio of 0.57 and no neff ratios below 0.29. Rhat values show good convergence with no Rhat values above 1.0106 for model parameters (Table S1).

### Stress response factor calculation

To compare stress *responses* between genotypes, we calculated a stress response factor, accounting for inherent phenotypic differences between genotypes under control conditions. This stress response for each trait was calculated as the treated plant measurement relative to the control measurement. For hue, we calculated the circular difference as the change of degrees between the treatment and control divided by 180 because hue is a circular trait (i.e., 0 and 180 are neighboring values). For height and plant area, we calculated a percentage of the control (treatment divided by control multiplied by 100), where 100% describes no change in phenotype due to the treatment.

### Multiplicative risk model

A multiplicative risk model was used to determine if the effect of the combined stress on each phenotype was additive, antagonistic, or synergistic. First, the independent effect sizes of the drought, heat, and combined‐stress treatments on each trait at the last day of the experiment were calculated. Analyses of variance (ANOVA) followed by *post hoc* Tukey pairwise comparisons of means were run to calculate the difference between each treatment and the control group. The effect of each treatment was calculated as the treatment's difference from control over the mean of the control condition. The effect variance is calculated from the 95% confidence interval of the comparison of means.

$${\text{Effect}}_{\mathrm{gt}}=\frac{\underline{{\text{Treatment}}_{\mathrm{gt}}}-\underline{{\text{Control}}_{\mathrm{gt}}}}{\underline{{\text{Control}}_{\mathrm{gt}}}}$$

Next, a multiplicative risk model was used to estimate a predicted additive effect for the combined stress as the sum of the independent effects of drought and heat ($D_{\text{effect}}$ and $H_{\text{effect}}$) minus their product. In comparison to a simpler additive model, the multiplicative risk model corrects over‐inflated calculations, to avoid unrealistic effects such as reductions larger than 100% of the control group.

$$\text{ExpectedDH}=\left(D_{\text{effect}}+H_{\text{effect}}\right)-\left(D_{\text{effect}}*H_{\text{effect}}\right)$$

The variance of this predicted combined‐stress effect is calculated using the variances of the independent drought and heat effects using the delta method, as suggested by Darling et al. (2010).

$${\mathrm{var}}_{\text{ExpectedDH}}={\mathrm{var}}_{D}*{\left(1-H_{\text{effect}}\right)}^{2}+{\mathrm{var}}_{H}*{\left(1-D_{\text{effect}}\right)}^{2}$$

To determine the effect of the combined stress, the observed effect of the combined stress is subtracted from the predicted combined effect calculated from the multiplicative risk model. Comparing their confidence intervals determines the type of effect the stress combination has on each trait. If the confidence interval of the subtraction overlaps zero, the effect of the combined stress is considered additive. If the subtraction results in a positive value and its confidence interval does not overlap zero, the effect will be synergistic. A negative value and no confidence interval overlap indicates an antagonistic effect (https://github.com/danforthcenter/maize‐abiotic‐stress‐paper‐2026).

## CONFLICT OF INTEREST

The authors declare no conflict of interest.

## Supporting information


**Figure S1.** Variance explained by genotype and treatment: (a) Variance explained by treatment, genotype, genotype × treatment, and residuals using a random‐effects model on the last day of the experiment. (b) Variance explained by treatment over the experimental period.

**Figure S2.** Correlation between traits: Correlation matrix for all traits extracted from PlantCV output.

**Figure S3.** Number of leaves: Leaf count per genotype at each treatment. Asterisks indicate significant differences from the genotype's respective control treatment as determined by Dunn's test pairwise comparison (*P* < 0.05).

**Figure S4.** Average water used over time by plants in each treatment: Plants were watered automatically based on daily gravimetric measurements and target weights. Water used by each plant was calculated as the difference in weight after watering each day and before watering the following day.

**Figure S5.** Response of plant height: Relative change in plant height across time for each treatment for all 47 genotypes.

**Figure S6.** Response of plant color: Relative change in plant color across time for each treatment for all 47 genotypes.

**Figure S7.** Response of plant pWUE: Relative change in pWUE across time for each treatment for all 47 genotypes.

**Figure S8.** Trait response by subpopulation. Similarity of stress phenotype relative to control. Chi‐squared and respective *P* value of log‐ratio tests to identify significant differences between subpopulations at the top of each subset.

**Figure S9.** Plot of growth curves from 200 simulated model prior draws for the power law model.

**Table S1.** Bayesian hierarchical model fit metrics and parameter estimates for all genotypes and treatments.

**Table S2.** Results of Bayesian hypothesis testing for increasing (*B* > 1) or decreasing (*B* < 1) growth rate. Star indicates significance.

**Table S3.** Spearman correlations between phenotype responses within and between treatments.

**Table S4.** Spearman correlations between control phenotype and phenotype response in each treatment.

**Table S5.** Results of Bayesian hypothesis testing for earliest significant difference in plant area.

**Table S6.** Correlations between phenotype responses and stress onset day in drought stress.

**Table S7.** Source information of the germplasm used in this study.

## Data Availability

The data that support the findings of this study are openly available in GitHub: https://github.com/danforthcenter/maize‐abiotic‐stress‐paper‐2026, and BioImage Archive: reference number 10.6019/S‐BIAD2511.
