# Supplementary material for: Temporal phenotypic responses of early vegetative maize to heat, drought, and combined stresses
Source: Plant J. 2026 Oct 10;128(1):e71161. doi: 10.1111/tpj.71161 (PMC13653223; doi:10.1111/tpj.71161)

Supplemental Figure 3

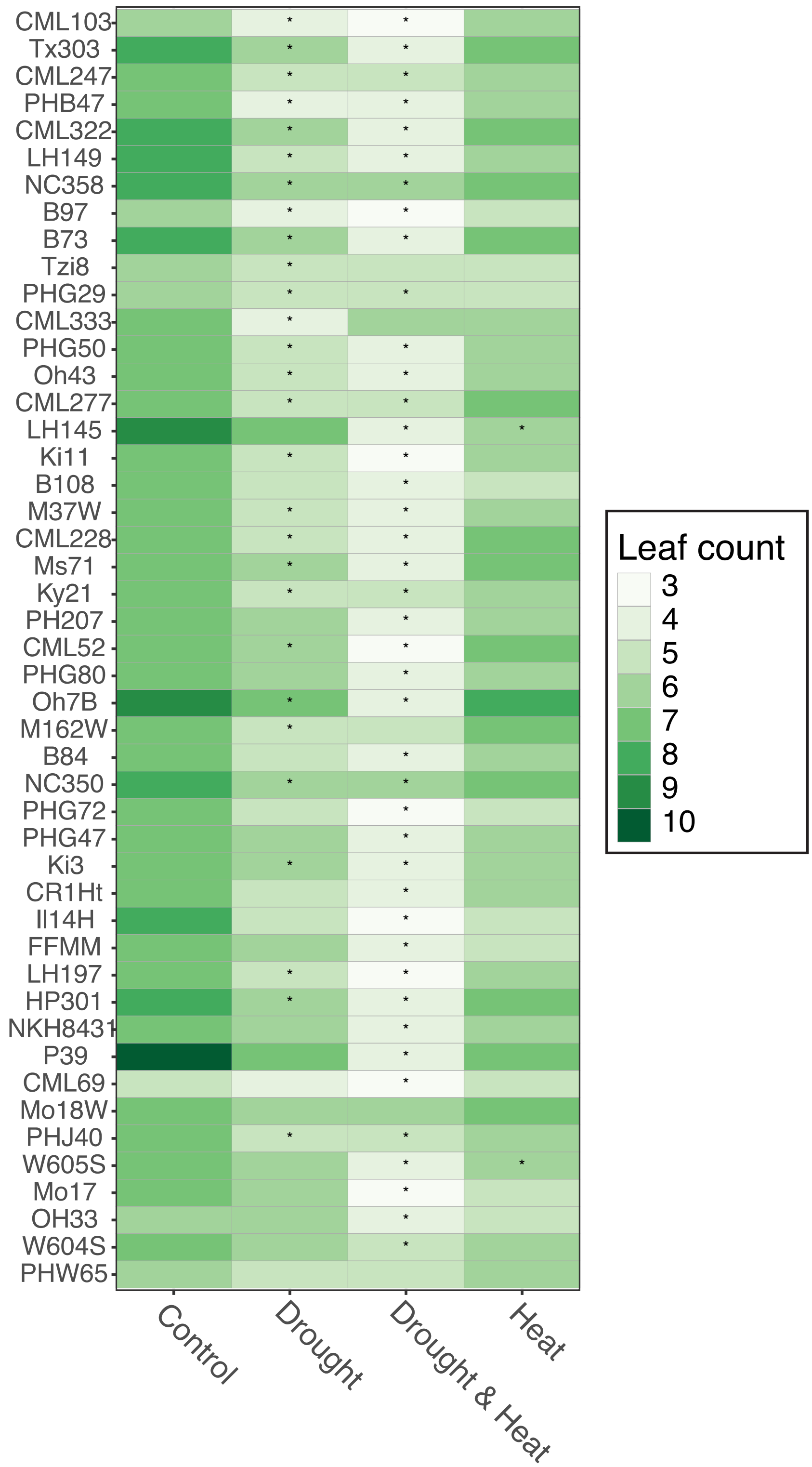

Supplement: Supplementary file 2 [file TPJ-128-0-s005.pdf]
